# The influence of sleep disruption on learning and memory in fish

**DOI:** 10.1111/jsr.70005

**Published:** 2025-03-19

**Authors:** Will Sowersby, Taiga Kobayashi, Satoshi Awata, Shumpei Sogawa, Masanori Kohda

**Affiliations:** ^1^ Laboratory of Animal Sociology, Department of Biology Osaka Metropolitan University Osaka Japan; ^2^ Present address: Arthur Rylah Institute for Environmental Research Heidelberg Australia

**Keywords:** cognition, evolution, fish, learning, memory, sleep

## Abstract

Sleep is a ubiquitous process that has been conserved in animals. Yet, our understanding of the functions of sleep largely derives from a few species. Sleep is considered to play an important role in mental processes, including learning and memory consolidation, but how widespread this relationship is across taxa remains unclear. Here, we test the impact of sleep disruption on the ability of the cleaner fish (*Labroides dimidiatus*) to both learn and remember a novel cognitive task. Sleep was disrupted by exposing a subset of fish to light at set intervals during the night. We found a significant negative relationship between sleep disruption and the ability to learn a novel task. Specifically, we found that fish in the light‐disturbed sleep treatment took significantly longer and made more incorrect decisions to find a food reward, compared with the undisturbed sleep treatment. All fish were then allowed a normal sleep schedule and retested several days later to assess their ability to remember the task. In contrast to the learning phase, we observed no significant differences between the two treatment groups in remembering the food reward several days later. Our results demonstrate a negative impact of sleep disruption on performance in a cognitive challenging task that appeared to have the strongest effect when fish were first exposed to the challenge. Importantly, we show that the association between sleep and mental processes, such as learning, may be widespread across vertebrate taxa and potentially have an early origin in the evolutionary history of vertebrate animals.

## 1 INTRODUCTION

Sleep is a ubiquitous trait that has been conserved across animal taxa, with positive links to immune function, neurogenesis, wellbeing, and cognitive performance (Tononi & Cirelli, 2006; Elvsåshagen et al., 2015). There are also costs associated with sleeping, such as potentially being more vulnerable to predators and having reduced time available to secure food and mates (Lima, 1998; Lima et al. 2005; Joiner, 2016; Tworkowski & Lesku 2019). Nevertheless, there is currently no evidence to suggest that animals can completely renounce sleep without detrimental consequences, including death (Van Dongen et al. 2003; Cirelli & Tononi, 2008). Indeed, sleep is maintained homeostatically with a commonly observed response to sleep disruption being longer and deeper sleep (Nath et al., 2017; Tobler, 2011; Tobler & Borbély, 1985; Yokogawa et al., 2007). Despite seemingly strong selective pressure to conserve sleep in animals, how sleep has coevolved with other traits and processes is still not commonly considered in evolutionary research (Cirelli & Tononi, 2008; Mignot, 2008; Rattenborg et al., 2017; Roth et al., 2010).

One exciting, but still controversial proposed function of sleep is the important role it plays in brain processes. For example, compelling evidence points to a strong positive link between sleep, learning, and memory consolidation (Huber et al., 2004; Walker & Stickgold, 2004, 2006; Peigneux et al., 2001; Smith, 2001; Stickgold, 2006; Anafi et al., 2019; Siegel, 2001; but see Vertes, 2004; Rattenborg et al., 2017), neural maintenance (Kavanau, 1996; Tononi & Cirelli, 2006), and cognitive ability (Scullin & Bliwise, 2015; Wagner et al., 2004). In animals, learning and memory are critical cognitive functions for a range of tasks, including foraging or food‐caching (Sherry & Hoshooley, 2010; Shettleworth, 1995), navigation and territory maintenance (Shettleworth, 1998), and for recalling positive and negative social interactions (Mateo & Johnston, 2000). The link between sleep and memory consolidation has been demonstrated in humans (Born et al., 2006; Diekelmann & Born, 2010; Huber et al., 2004), other mammals (Capellini et al., 2009; Ribeiro et al., 2004; Smith & Kelly, 1988; Walker & Stickgold, 2004), and to a lesser extent in birds (Derégnaucourt et al., 2005; Jackson et al., 2008). Nevertheless, research investigating the associations between sleep and learning/memory has largely been taxonomically limited (Keene & Duboue, 2018) despite the evidence that sleep may play an important role in memory formation in invertebrates (e.g. Beyaert et al., 2012; Krishnan et al., 2016) and fishes (e.g. Pinheiro‐da‐Silva et al., 2018; Rawashdeh et al., 2007).

Several functions and structures of the vertebrate brain, especially the forebrain, appear to have been largely conserved throughout the evolution of vertebrate taxa (Vargas et al., 2006; Xie & Dorsky, 2017). For example, the mammalian and avian hippocampus, along with the homologous pallial areas of the reptile and fish brain share a central role in supporting and encoding spatial information (Rodríguez et al. 2002; Portavella et al., 2002). Vargas et al. (2006) demonstrated that lesions to the lateral pallium in goldfish (*Carassius auratus*) can impair the encoding of geometric spatial information, comparable to the effect of lesions on the hippocampus in mammals and birds. Likewise, patterns of sleep also appear similar across taxonomic groups, including in fishes, such as the observation of characteristic sleep behaviours (e.g. postural relaxation and reduced responsiveness; in cartilaginous fishes, Kelly et al., 2019, 2022) and deep slow‐wave and rapid eye like movements (REM; in bony fishes: Leung et al., 2019; M. Kohda et al. pers. comms.). Evidence demonstrating an important role of sleep for learning and active memory consolidation in non‐mammalian species would imply that sleep has played a key role in these mental processes across vertebrate evolutionary history (Vorster & Born, 2015). Links between sleep, learning and memory in fishes and other non‐mammalian taxa is, however, not as well established (Pinheiro‐da‐Silva et al., 2018; Rawashdeh et al., 2007) and this large and diverse group of vertebrates has relatively been neglected from studies on sleep (Campbell & Tobler, 1984; Norman et al., 2024, but see Moorman et al., 2015; Johnsson et al., 2022).

Here, we investigate the impact of sleep disruption on learning ability and subsequent memory consolidation in the bluestreak cleaner wrasse (cleaner fish; *Labroides dimidiatus*). This fish species has a wide distribution on coral reefs across the Indo‐Pacific, where in a mutualistic relationship, it eats parasites and dead tissue off ‘client’ species. Hence, this highly social fish, regularly engages in cognitively challenging tasks, such as recalling both favourable and antagonistic interactions with conspecifics and numerous client individuals from a range of different species. Cleaner fish are highly amenable to captive experimental conditions and have demonstrated several complex cognitive abilities, some which may be impacted by sleep quality (e.g. Bshary & Brown 2014; Kohda et al. 2019, 2023). Cleaner fish are diurnal and shelter in caves or crevices overnight where they remain motionless until the morning (Lenke, 1988). We predict that when subjected to disrupted sleep schedules, cleaner fish will demonstrate poorer performance in novel cognitively challenging tasks, in comparison with fish that have experienced undisrupted, normal sleep regimes. We also predict that cleaner fish subjected to disruptions in normal sleep regimes will take longer to rouse each morning and react slower to the presence of food, compared with fish that have experienced undisrupted sleep.

## 2 METHODS

### 2.1 Animal husbandry

Cleaner fish (*n* = 12) were purchased from reputable tropical fish specialists and were individually housed (aquaria 40 L) under laboratory conditions (14:10 h light regime; 27°C) at the Department of Biology, Osaka Metropolitan University, Japan. All aquaria were furnished with coral sand substrate and a small opaque piece of PVC pipe to provide shelter. Water changes and water parameter checks (including salinity) were conducted regularly. Fish were fed daily with commercially bought shrimp (Pandalidae), mashed into a paste and suspended in the tank on a grey plastic plate. A pilot trial of the experimental set‐up took place in early 2020, with the main experiment then conducted between September and October 2023. All experiments adhered to the animal ethics guidelines of Osaka Metropolitan University.

### 2.2 Sleep treatment

**FIGURE 1:**
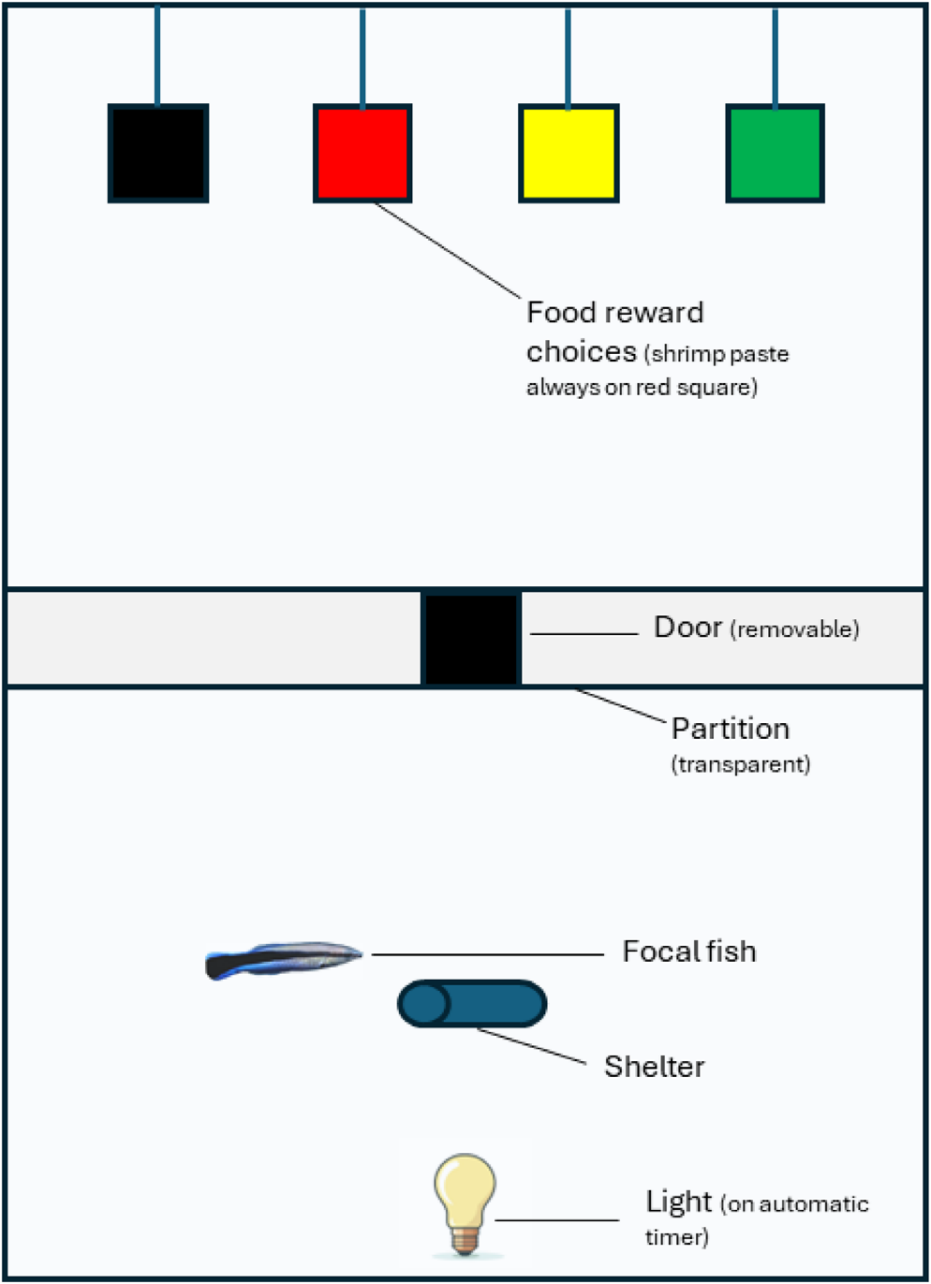
Simplified top‐down view of the experimental set‐up to test the effect of sleep disruption on the ability to learn and remember a food reward.

Fish were moved into their treatment tanks for a 48‐h acclimation period prior to the start of the experiment. Treatment tanks were identical to the housing aquaria, except the treatment tanks were divided into two by a clear plastic barrier with a door and had a lamp overhead (Figure 1). Fish were secured in the back half of the tank and were provided with food in the front half of the tank, once per day. Specifically, shrimp mashed into a paste was put on a grey plastic square which was hung into the front half of the tank. When the door in the partition was raised the fish could enter the front half of the tank and became accustomed to accessing that half of the tank via the door to feed. After 3 h the grey plastic square was removed, and fish were gently ushered back into the second half of the tank if they had not already returned voluntarily.

After the acclimation period, half of the fish (*n* = 6) were assigned to the light‐disturbed sleep treatment and the remaining half to the undisturbed sleep treatment (*n* = 6). Fish in the undisturbed sleep treatment were kept in complete darkness for a period of 10 h (22:00–8:00) each night, a period which corresponds to the typical night length in the tropical regions where these fish naturally occur. In contrast, fish in the light‐disturbed sleep treatment were exposed to a light (colour: daylight white/white, 4.5 W, 672 lux) above the tank that automatically turned on every 2 h, for a period of 1 h (controlled by a timer), meaning that fish in this treatment were exposed to fewer periods of darkness per night, broken into 2‐h blocks. Using light to suppress sleep has successfully been used in zebrafish research (Yokogawa et al., 2007). Moreover, the opaque pipe shelter was removed during the light‐disturbed sleep treatment stage and replaced with a transparent plastic pipe of identical size, which would provide the fish with a secure place to shelter, but not offer refuge from the light above.

To prevent fish in the undisturbed sleep treatment from being disturbed by light during the night, fish in the light‐disturbed sleep treatment were isolated at night via a black‐out sheet. Camcorders (SONY Handycam HDR‐CX480) positioned in front of each tank (*n* = 4) were quietly switched on 10 min prior to the morning light coming on (at 8:00). The room was blacked out to prevent natural light entering, and researchers entered using a dim red light. All fish were recorded for ~20 min after the morning light automatically switched on at 8.00 a.m, to capture the time it took each fish to wake up (i.e. when they left the shelter and started swimming), and their initial activity behaviour (i.e. time spent moving and distance travelled).

The sleep treatment occurred every night for four consecutive nights, but only during the learning phase of the experiment. It is important to note, during the subsequent rest and memory phases of the experiment all fish were exposed to darkness for the normal 10‐h period and were provided with an opaque tube for shelter (i.e. transparent tubes from the light‐disturbed sleep treatment tanks were replaced with opaque tubes). A subset of the fish from both treatments were recorded at night throughout the learning phase of the experiment, confirming that fish in the light‐disturbed sleep treatment were awakened and active when the light came on.

### 2.3 Food reward choice: Learning phase

We presented fish with a food reward choice at ~9.00 a.m. each morning, for 3 days, beginning from the first morning after the night of the first sleep treatment. Each fish was presented with a row of four plastic squares, each a different colour (red, green, black, and yellow) suspended in a row in the front half of the tank (Figure 1). Shrimp paste had been placed on the back of only the red square, so fish were tasked with finding the food on the back of this square. Each morning for the 3 days, fish were presented with the same four coloured squares in a row, always in a different order to previous days. Once the squares were in place the door separating the front and back halves of the tank was lifted and we recorded the following, (1) time to exit the door, (2) number of incorrect choices, (3) time to find the food on the back of the red square. We considered an incorrect choice to be any deliberate touching with the mouth or head of a square that was not the backside of the red square. After three mornings, fish in the light‐disturbed sleep treatment were exposed to a final night of disrupted sleep before all fish entered a 6‐day rest period under normal sleep conditions.

### 2.4 Food reward choice: Memory phase

After the rest period, the fish were again exposed to the same food reward choice experiment for another 3 days. In contrast to the learning phase and like the rest period, no fish were exposed to light during the night in this phase of the experiment. This component of the experiment tested the ability of the cleaner wrasse to recall the correct location of the food reward on the red coloured square. As previously, we recorded, (1) time to exit the door, (2) number of incorrect choices, (3) time to find food on the back of the red square. After the third day each fish was weighed and photographed and placed back in its home tank.

### 2.5 Statistical analysis

We performed separate linear mixed models (lmm; R packages lme4 and lmerTest) to test the effects of light‐disruption on rousing time, time to exit door, and time to correct choice. In each model we included the variables sleep treatment (light‐disrupted or undisrupted), day (day 1, 2, or 3), and their interaction, with fish ID added as a random effect. We also performed a generalised linear mixed model (glmm; Poisson distribution; R packages lme4 and lmerTest) to test the effects of light‐disruption on the number of incorrect choices. Likewise, we included the variables sleep treatment (light‐disrupted or undisrupted), day (day 1, 2, or 3), and their interaction, with fish ID added as a random effect. Significant effects were determined using the Anova function (R package car, type II). Pairwise comparisons of the estimated marginal means in LMMs and GLMMs were conducted using the emmeans function (R package emmeans). For each main treatment effect reported in the text we also calculated effect sizes (partial) etasqu for lmm models (R package effectsize) and Incidence Rate Ratios (IRRs) for glmm models. The effects of light‐disruption on time to exit door, time to correct choice, and number of incorrect choices were analysed in both the learning and memory phases. All statistical analyses were conducted in R v4.2.1 (R Core Team, 2022).

## 3 RESULTS

### 3.1 Impact of light‐disruption on ability to learn cognitive task

In contrast to our first prediction, we did not observe any significant difference in the time to first movement between fish in the light‐disturbed and undisturbed sleep treatments (rousing time; LMM, treatment: df = 1, chisq = 0.005, p = 0.94, partial etasq = 0.001; treatment x day: df = 3, chisq = 1.91, p = 0.59, partial etasq = 0.07; Supplementary Table S1). On average, fish in the light‐disturbed sleep treatment first moved from their shelter after 197 s and undisturbed treatment fish 220 s once the light automatically turned on at 8.00 a.m.

On day 1 of the learning phase of the experiment, undisturbed sleep fish exited the door significantly quicker (mean 101.0 s) than the light‐disturbed fish (mean 1152.3 s; emmeansm df = 30, *t‐ratio* = 2.12, *p* = 0.042, etasq = 0.13; Supplementary Statistics 1, but also see Supplementary Table S2). The time it took fish in both sleep treatments to exit the door reduced with each subsequent day (Supplementary Table S2).

**FIGURE 2:**
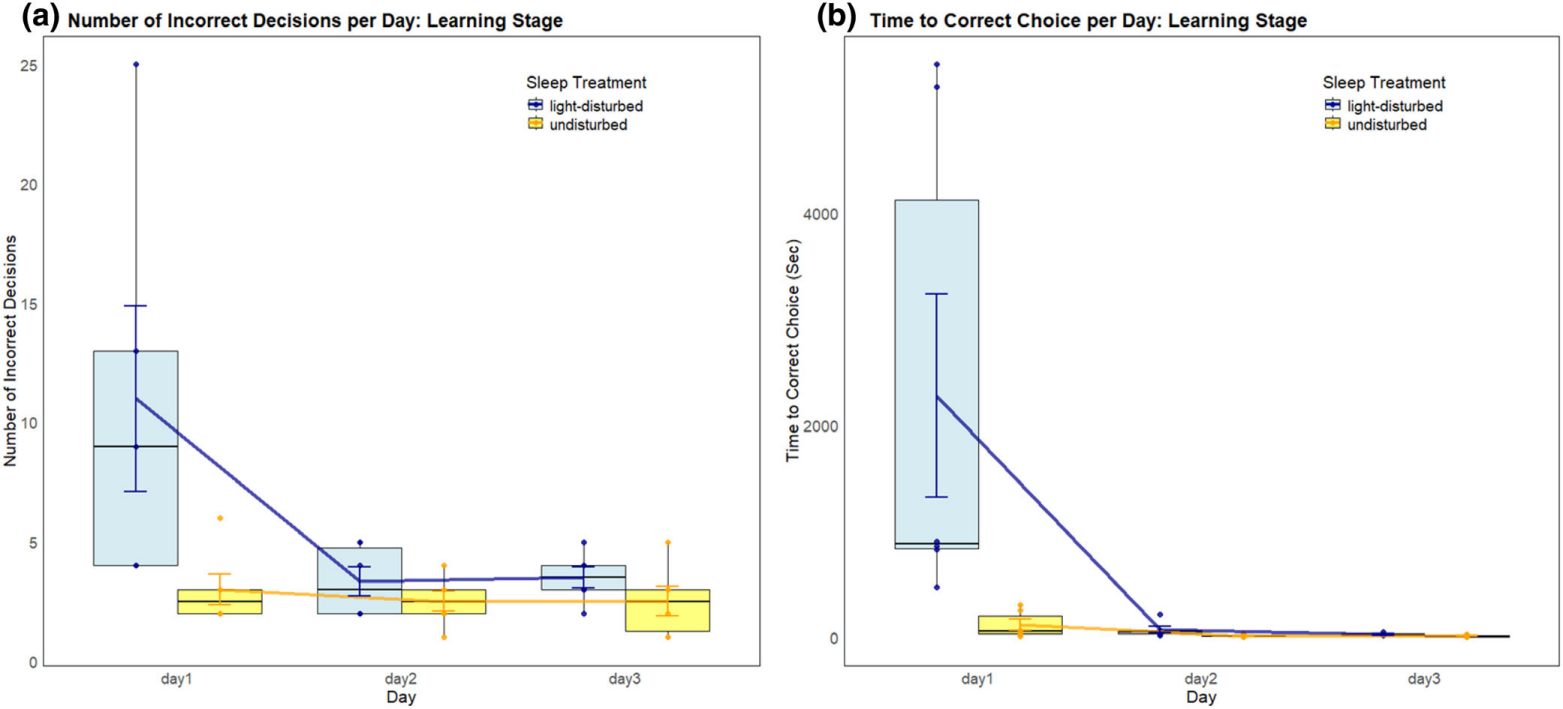
(a) The mean number of incorrect decisions made by cleaner fish in two different sleep treatments (orange: Undisturbed; blue: Light‐disturbed) to find a food reward during the learning phase of the experiment. Mean incorrect choices per day; day 1: Light‐disturbed fish 11.0, undisturbed 3.0; day 2: Light‐disturbed fish 3.3, undisturbed 2.5; day 3: Light‐disturbed fish 3.5, undisturbed 2.5. (b) The mean time taken to find the food reward. Mean time per day; day 1: Light‐disturbed fish 2277.7 s, undisturbed 120.5 s; day 2: Light‐disturbed fish 74.5 s, undisturbed 17.7 s; day 3: Light‐disturbed fish 29.7 s, undisturbed 16.8 s. In both, the boxplots display the treatment means and higher/lower quartiles, the line represents the trend over time, whiskers show the standard errors, and the coloured points display individual values. See Supplementary Statistics 2 and 3 for pairwise comparisons.

In line with our second prediction, we did find a significant negative effect of light‐disruption on the ability to find the correct location of the food reward (Poisson GLMM, treatment x day: df = 2, chisq = 7.72, p = 0.02, IRR = 0.74; Figure 2a; Supplementary Table S3). Specifically, on day 1, fish in the light‐disturbed sleep treatment performed on average significantly more incorrect choices before finding the food reward (11 incorrect choices) compared with fish in the undisturbed sleep treatment (3 incorrect choices; emmeans, df = Inf, z‐ratio = 3.98, p = 0.0001; Figure 2a, Supplementary Statistics 2). As the days progressed, the mean number of incorrect choices by both treatment groups declined (Figure 2a).

Similarly, we found a significant negative effect of light‐disruption on the time to find the correct food reward square (LMM, treatment x day: df = 2, chisq = 10.07, *p* = 0.006, partial etasq = 0.33, Figure 2b; Supplementary Table S4). On day 1, Light‐disturbed fish took on average 2277.7 s to find the food reward, while undisturbed fish took 120.5 (emmeans, df = 30, t‐ratio = 3.90, p = 0.0005, etasq = 0.34; Figure 2b; Supplementary Statistics 3). Again, fish in both groups improved over time (Figure 2b).

### 3.2 Impact of past light‐disruption on the ability to remember a cognitive task

In contrast to the learning phase, we found no significant difference in the mean time to exit the door between the two treatments after the rest phase of the experiment (LMM, treatment: df = 1, chisq = 0.62, *p* = 0.43, partial etasq = −0.06; treatment x day: df = 2, chisq = 1.24, p = 0.54, partial etasq = 0.06; Supplementary Table S5).

**FIGURE 3:**
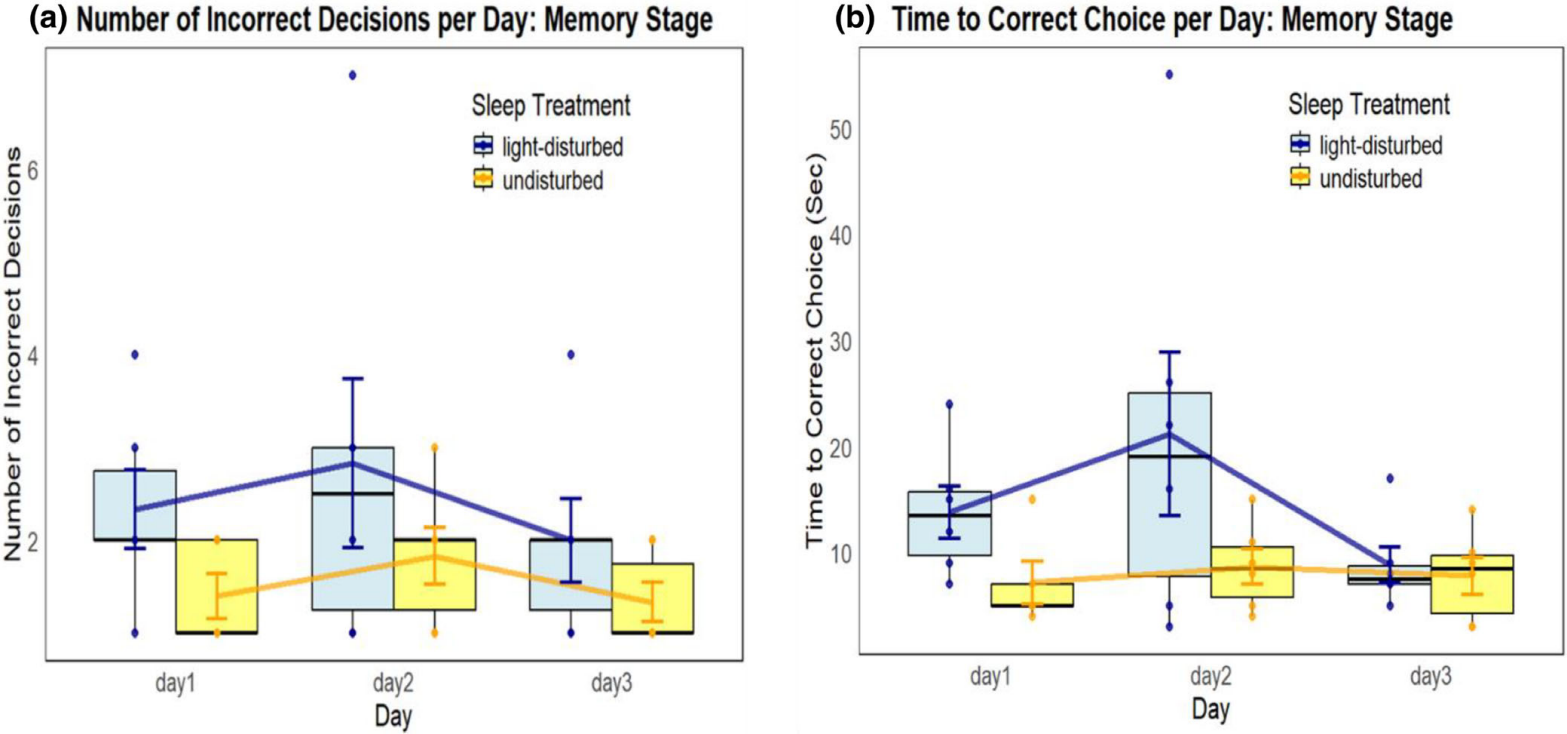
(a) The mean number of incorrect decisions made by cleaner fish in two different sleep treatments (orange: Sleep disrupted; blue: Sleep not disturbed) to find a food reward during the memory phase of the experiment. Mean incorrect choices per day; day 1: Sleep disturbed fish 2.3, non‐disturbed 1.4; day 2: Sleep disturbed fish 2.8, non‐disturbed 1.8; day 3: Sleep disturbed fish 2.0, non‐disturbed 1.3. (b) The mean time taken to find the food reward in two different sleep treatments during the memory phase of the experiment. Mean time per day; day 1: Sleep disturbed fish 13.8 s, non‐disturbed 7.2 s; day 2: Sleep disturbed fish 21.2 s, non‐disturbed 8.7 s; day 3: Sleep disturbed fish 8.8 s, non‐disturbed 7.8 s. In both, the boxplots display the treatment means and higher/lower quartiles, the line represents the trend over time, whiskers show the standard errors, and coloured points display the individual values. See Suplementary Statistics 4 and 5 for pairwise comparisons.

During the memory phase, we also did not find a significant difference between the sleep treatments in the mean number of incorrect choices made before finding the food reward (Poisson GLMM, treatment: df = 1, chisq = 2.61, *p* = 0.1, IRR = 0.7; treatment x day: df = 2, chisq = 0.03, p = 0.99; Figure 3a; Supplementary Table S6; Supplementary Statistics 4). Individuals that had been sleep disrupted during the previous learning phase performed on average 2.4 incorrect choices, while non‐disturbed sleep individuals performed on average 1.5 incorrect choices.

Likewise, overall, we found no significant difference in the time to find the food reward (LMM, treatment: df = 1, chisq = 2.85, *p* = 0.09, partial etasq = 0.22; treatment x day: df =2, chisq = 4.06, p = 0.13, partial etasq = 0.17; Figure 3b; Supplementary Table S7). However, on day 2, individuals that had been sleep disrupted during the previous learning phase took on average 21.2 s, whereas non‐disturbed sleep individuals took on average 8.7 s, to find the food reward (emmeans, df = 22.7, t‐ratio = 2.43, p = 0.02, etasq = 0.21; Figure 3b; Supplementary Statistics 5).

In both groups, individuals performed better at the food reward task in the memory phase compared with the learning phase. Generally, performances continued to get better as the memory phase progressed, however, the pattern was not as clear as the continued improvement in completing the task in the learning phase.

## 4 DISCUSSION

We clearly demonstrate that disruptions to a normal sleep pattern can negatively affect how quickly and accurately an animal performs a novel cognitive task. We used as our model species the cleaner fish, to provide evidence of the important functions of sleep in a non‐mammalian and non‐avian species. Despite retaining small mean differences over time, we did not observe any significant effect of sleep treatment on the ability to perform the cognitive task during the memory phase of the experiment. Indeed, the ability to perform the task continued to improve in both treatment groups throughout both the learning phase and to a lesser extent the memory phase of the experiment. Under our experimental conditions, the negative effects of sleep disruption seem to be most apparent when first encountering a new cognitive challenge. Nevertheless, our results support the claim that sleep plays an important role in mental processes, such as learning, across vertebrate taxa.

The ability to learn is a critical function allowing strategic adaptation to changing environmental demands. Cleaner fish inhabit structurally complex physical environments and dynamic social environments and to be successful they require the ability to learn and to adapt their behavioural responses to changes accordingly. For example, it is estimated that wild cleaner fish remove up to 1200 ectoparasites each day from anywhere between 800 to 3000 client fish (Grutter, 1995, 1999; Wismer et al., 2019). In this context, cleaner fish must learn and remember information about their clients, including past positive or negative interactions, and identifying which clients are residents or visitors to their cleaning territories. We know that cleaner fish are also capable of social learning, with juvenile fish learning strategies to interact with clients by observing adult cleaner fish (Truskanov et al. 2020). We also know that cleaner fish are not the only fish species capable of learning, for instance archer fish (*Toxotes* sp.) learn complex skills from observing other group members (Schuster et al., 2006), while guppies are capable of learning and anticipating the path of a mechanical fish (Bierbach et al., 2022) and can successfully complete reverse learning choice tasks (Buechel et al., 2018). It is therefore likely that other fish will also be negatively impacted by sleep disruption when learning novel tasks.

Sleep is predicted to maintain optimal brain functioning to support waking cognition (Alhola & Polo‐Kantola 2007). In cleaner fish, the negative effects of sleep disruption were most apparent at the beginning of the learning phase of the experiment. Our results may suggest differences in the timing of the performance of operant behaviours, i.e. those that produce consequences and may be repeated if they provide a reward (Toates, 2012), with sleep‐interrupted fish taking longer to find the food reward on the first day after sleep disruption. An explanation for our finding could be that fish in the light‐disrupted sleep treatment are less active or motivated. Indeed, a similar finding has been observed in Australian magpies, where sleep loss impaired motivation and cognitive performance (Johnsson et al., 2022). In contrast, we found that sleep disruption appeared to have less of an effect on reinforcement learning on subsequent days, with the time taken to find the food reward similar between both sleep treatments on days 2 and 3 of the experiment. Reinforcement learning influences behaviour via either a positive or negative feedback response and can be somewhat affected by sleep loss (Gerhardsson et al., 2020; Whitney et al., 2015). There also remains the possibility that fish in the light‐disrupted sleep treatment became habituated to the experimental set‐up over time. In any case, we recommend that future experiments investigating the association between sleep and learning should include a reverse learning element, which has been shown in humans to amplify the impairment caused by sleep loss (Whitney et al., 2015).

We did not find a significant negative effect of sleep disruption on the ability of cleaner fish to recall the food reward cue after several days post learning phase (with the exception of day 2). In humans, memory functions comprise three major subprocesses, i.e. encoding, consolidation, and retrieval (Straube, 2012). Research has indicated that sleep after learning can have an important influence on memory consolidation. Rather we found that the performance of fish in both sleep treatments increased with time and exposure to the task in both the learning and to a lesser extent the memory stages of the experiment. Our findings do not rule out an important role for sleep in memory consolidation in fish, specifically non‐declarative memory, but potentially represents a limitation in our experimental design. For example, the cognitive task may have been too simple or was repeated too often during the experimental period. Another possible explanation is that our periods of sleep disruption during the learning phase were too short to affect the consolidation and retrieval of memories associated with reinforcement learning. For instance, Pinheiro‐da‐Silva et al. (2017, 2018) found that total sleep deprivation, but not the partial, impacted learning and memory performance in zebrafish. In our experiment the fish were still allowed several hours sleep each night during the learning stage, albeit broken into discreet periods of time by the automated light and had returned to a normal sleep schedule by the memory stage. It is possible that a more complex cognitive task, including a reverse learning component, longer periods of sleep deprivation each night, or continuing the light‐disruption sleep treatment throughout the memory stage, may have increased cognitive and recall impairment.

The involvement of sleep in memory processing has been investigated for at least a century (Jenkins & Dallenbach, 1924; Rasch & Born, 2013). Yet, sleep research is still overwhelmingly biased towards humans, other mammals, and laboratory model species (Toth & Bhargava 2013; Blumberg et al. 2020). Experimental evidence has demonstrated behavioural, anatomical, genetic, and pharmacological conservation of sleep between fish (evidence largely comes from zebrafish research) and mammals, suggesting that research in fish can help to inform our understanding of mammalian sleep (Lee et al., 2022). Despite difficulties finding neuronal sleep signatures in fishes due to the absence of a conventional neocortex, bony fishes do possess a homologue of the dorsal pallium, and neural signatures of sleep have been observed (Leung et al., 2019). Our evidence that sleep has an impact on mental processing in fish, in combination with the conservation of sleep and the presence of homologous structures in the vertebrate brain, suggests that sleep has played an important role in animal cognitive processes since early in the evolutionary history of vertebrate taxa.

In humans, night exposure to light can disrupt circadian rhythms, affecting sleep as well as other psychological factors such as motivation, stress responses, and cognitive function (Burns et al., 2023). We found clear results that a light‐disturbed sleep treatment negatively affected cognitive function in the cleaner fish. While we interpret our results as demonstrating an effect of sleep disruption on learning ability, we also speculate that stress and motivation may have additionally influenced our results. Uncoupling these factors would prove difficult as lack of sleep is a stress inducing experience and can also reduce motivation. One possibility could be to have an opaque shelter in the sleep disturbed treatment group, instead of a transparent shelter, to provide fish with a more secure feeling shelter. However, it is untested whether having an opaque shelter would reduce the effectiveness of the light to disrupt sleep. Importantly, we did observe the light causing fish to become active overnight, a similar observation to Yokogawa et al. (2007). Yokogawa and colleagues observed that light had a greater suppressive effect on sleep than electrical stimulation and was stronger than circadian influences. Previous studies have also found that light suppresses rest in other teleosts (Tobler & Borbély, 1985) and that artificial light stops the display of natural activity and circadian cycles, for example, in wild rockfish (*Girella laevifrons*; Pulgar et al., 2019). These studies suggest that light or melatonin overcome other networks regulating sleep in fishes (Yokogawa et al., 2007; Zhdanova et al., 2001) providing confidence in the use of light as a sleep suppressant in our study.

Our study provides further evidence that sleep is functionally important in non‐mammalian animals. We found that the negative impacts of sleep disruption were most apparent initially, likely impacting cognition, activity levels, and motivation, which in turn slowed the learning process. Our results do not uncover which aspects of cognition were most affected by sleep loss. However, we speculate that the association between sleep and mental processes is widespread in vertebrates and may have coevolved or facilitated the ability of animals to perform complex cognitive behaviours.

## AUTHOR CONTRIBUTIONS

**Will Sowersby:** Conceptualization; methodology; writing – original draft; formal analysis; data curation; investigation. **Taiga Kobayashi:** Methodology; writing – review and editing. **S. Awata:** Formal analysis; resources; writing – review and editing; supervision. **Shumpei Sogawa:** Resources; project administration; writing – review and editing; methodology. **Masanori Kohda:** Conceptualization; writing – original draft; funding acquisition; project administration; resources; supervision; methodology.

## Supporting information


**Table S1.** Time to rousing (Learning Phase).
**Table S2.** Number of incorrect choices before food reward (Learning Phase).
**Table S3.** Time to correct choice (Learning Phase).
**Table S4.** Number of incorrect choices before food reward (Memory Phase).
**Table S5.** Time to correct choice (Memory Phase).

## Data Availability

The data that support the findings of this study are available from the corresponding author upon reasonable request.
