# Supplementary material for: The influence of sleep disruption on learning and memory in fish
Source: J Sleep Res. 2025 Mar 19;34(6):e70005. doi: 10.1111/jsr.70005 (PMC12592841; doi:10.1111/jsr.70005)
Supplement: Supplementary file 1 — Table S1. Time to rousing (Learning Phase). Table S2. Number of incorrect choices before food reward (Learning Phase). Table S3. Time to correct choice (Learning Phase). Table S4. Number of incorrect choices before food reward (Memory Phase). Table S5. Time to correct choice (Memory Phase). [file JSR-34-e70005-s001.docx]

**Supplementary Material**

**Table 1.** Analysis of deviance table for linear mixed model (LMM) fitted for time to rousing (Learning Phase).

| **Factor** | **Chisq** | **Df** | **Pr(>Chisq)** |
| --- | --- | --- | --- |
| Sleep treatment | 0.005 | 1 | 0.942 |
| Day | 2.259 | 3 | 0.520 |
| Sleep treatment x Day | 1.911 | 3 | 0.591 |

Type II Wald chi-square tests were performed.

**Table 2.** Analysis of deviance table for linear mixed model (LMM) fitted for time to exiting the door (Learning Phase).

| **Factor** | **Chisq** | **Df** | **Pr(>Chisq)** |
| --- | --- | --- | --- |
| Sleep treatment | 1.552 | 1 | 0.213 |
| Day | 4.111 | 2 | 0.128 |
| Sleep treatment x Day | 2.961 | 2 | 0.228 |

Type II Wald chi-square tests were performed.

**Table 3.** Analysis of deviance table for Poisson generalised linear mixed model (GLMM) fitted for number of incorrect choices before food reward (Learning Phase).

| **Factor** | **Chisq** | **Df** | **Pr(>Chisq)** |
| --- | --- | --- | --- |
| Sleep treatment | 8.233 | 1 | 0.004 |
| Day | 24.557 | 2 | <0.001 |
| Sleep treatment x Day | 7.721 | 2 | 0.021 |

Type II Wald chi-square tests were performed.

**Table 4.** Analysis of deviance table for linear mixed model (LMM) fitted for time to correct choice (Learning Phase).

| **Factor** | **Chisq** | **Df** | **Pr(>Chisq)** |
| --- | --- | --- | --- |
| Sleep treatment | 5.117 | 1 | 0.024 |
| Day | 12.127 | 2 | 0.002 |
| Sleep treatment x Day | 10.072 | 2 | 0.006 |

Type II Wald chi-square tests were performed.

**Table 5.** Analysis of deviance table for linear mixed model (LMM) fitted for time to exiting the door (Memory Phase).

| **Factor** | **Chisq** | **Df** | **Pr(>Chisq)** |
| --- | --- | --- | --- |
| Sleep treatment | 0.620 | 1 | 0.431 |
| Day | 1.840 | 2 | 0.399 |
| Sleep treatment x Day | 1.239 | 2 | 0.538 |

Type II Wald chi-square tests were performed.

**Table 6.** Analysis of deviance table for poisson generalised linear mixed model (GLMM) fitted for number of incorrect choices before food reward (Memory Phase).

| Factor | Chisq | Df | Pr(>Chisq) |
| --- | --- | --- | --- |
| Sleep treatment | 2.611 | 1 | 0.101 |
| Day | 1.412 | 2 | 0.494 |
| Sleep treatment x Day | 0.025 | 2 | 0.988 |

Type II Wald chi-square tests were performed.

**Table 7.** Analysis of deviance table for linear mixed model (LMM) fitted for time to correct choice (Memory Phase).

| **Factor** | **Chisq** | **Df** | **Pr(>Chisq)** |
| --- | --- | --- | --- |
| Sleep treatment | 2.846 | 1 | 0.092 |
| Day | 5.490 | 2 | 0.064 |
| Sleep treatment x Day | 4.056 | 2 | 0.132 |

Type II Wald chi-square tests were performed.

**Supplementary Statistics 1.** Multiple comparisons using Tukey method for linear mixed model (LMM) fitted for time to exiting the door (Learning Phase). Results are on the log scale. Confidence level: 95%:

1. **Estimated Marginal Means (EMMs)**

| **Sleep Treatment** | **Day** | **Emmean** | **SE** | **df** | **Lower CL** | **Upper CL** |
| --- | --- | --- | --- | --- | --- | --- |
| Interrupted | 1 | 1152.3 | 350 | 30 | 437 | 1867 |
| Noninterrupted | 1 | 101.0 | 350 | 30 | -614 | 816 |
| Interrupted | 2 | 39.3 | 350 | 30 | -676 | 754 |
| Noninterrupted | 2 | 7.5 | 350 | 30 | -707 | 722 |
| Interrupted | 3 | 14.0 | 350 | 30 | -701 | 729 |
| Noninterrupted | 3 | 10.0 | 350 | 30 | -705 | 725 |

1. **Pairwise Comparisons (Sleep Treatment within Days)**

| **Day** | **Contrast** | **Estimate** | **SE** | **df** | **t-ratio** | **p-value** |
| --- | --- | --- | --- | --- | --- | --- |
| 1 | Interrupted - Noninterrupted | 1051.3 | 495 | 30 | 2.124 | 0.0420 |
| 2 | Interrupted - Noninterrupted | 31.8 | 495 | 30 | 0.064 | 0.9492 |
| 3 | Interrupted - Noninterrupted | 4.0 | 495 | 30 | 0.008 | 0.9936 |

1. **Pairwise Comparisons (Days within Sleep Treatment)**

| **Treatment** | **Contrast** | **Estimate** | **SE** | **df** | **t-ratio** | **p-value** |
| --- | --- | --- | --- | --- | --- | --- |
| Interrupted | Day1 - Day2 | 1113.0 | 490 | 20 | 2.269 | 0.0838 |
| Interrupted | Day1 - Day3 | 1138.3 | 490 | 20 | 2.321 | 0.0758 |
| Interrupted | Day2 - Day3 | 25.3 | 490 | 20 | 0.052 | 0.9985 |
| Noninterrupted | Day1 - Day2 | 93.5 | 490 | 20 | 0.191 | 0.9802 |
| Noninterrupted | Day1 - Day3 | 91.0 | 490 | 20 | 0.186 | 0.9812 |
| Noninterrupted | Day2 - Day3 | -2.5 | 490 | 20 | -0.005 | 1.0000 |

**Supplementary Statistics 2**. Multiple comparisons using Tukey method for Poisson generalised linear mixed model (GLMM) fitted for number of incorrect choices before food reward (Learning Phase):

1. **Estimated Marginal Means (EMMs)**

| **Sleep Treatment** | **Day** | **Emmean** | **SE** | **df** | **Lower CL** | **Upper CL** |
| --- | --- | --- | --- | --- | --- | --- |
| Interrupted | 1 | 2.367 | 0.193 | Inf | 1.989 | 2.750 |
| Noninterrupted | 1 | 1.053 | 0.270 | Inf | 0.524 | 1.580 |
| Interrupted | 2 | 1.150 | 0.259 | Inf | 0.642 | 1.660 |
| Noninterrupted | 2 | 0.871 | 0.289 | Inf | 0.304 | 1.440 |
| Interrupted | 3 | 1.199 | 0.255 | Inf | 0.700 | 1.700 |
| Noninterrupted | 3 | 0.871 | 0.289 | Inf | 0.304 | 1.440 |

1. **Pairwise Comparisons (Sleep Treatment within Days)**

| **Day** | **Contrast** | **Estimate** | **SE** | **df** | **z-ratio** | **p-value** |
| --- | --- | --- | --- | --- | --- | --- |
| 1 | Interrupted - Noninterrupted | 1.315 | 0.331 | Inf | 3.975 | 0.0001 |
| 2 | Interrupted - Noninterrupted | 0.279 | 0.387 | Inf | 0.722 | 0.4704 |
| 3 | Interrupted - Noninterrupted | 0.328 | 0.384 | Inf | 0.855 | 0.3927 |

1. **Pairwise Comparisons (Days within Sleep Treatment)**

| **Treatment** | **Contrast** | **Estimate** | **SE** | **df** | **z-ratio** | **p-value** |
| --- | --- | --- | --- | --- | --- | --- |
| Interrupted | Day1 - Day2 | 1.220 | 0.264 | Inf | 4.604 | <0.0001 |
| Interrupted | Day1 - Day3 | 1.170 | 0.260 | Inf | 4.496 | <0.0001 |
| Interrupted | Day2 - Day3 | -0.048 | 0.311 | Inf | -0.157 | 0.9865 |
| Noninterrupted | Day1 - Day2 | 0.182 | 0.348 | Inf | 0.525 | 0.8593 |
| Noninterrupted | Day1 - Day3 | 0.182 | 0.348 | Inf | 0.525 | 0.8593 |
| Noninterrupted | Day2 - Day3 | 0.000 | 0.363 | Inf | 0.000 | 1.0000 |

**Supplementary Statistics 3**. Multiple comparisons using Tukey method for linear mixed model (LMM) fitted for time to correct choice (Learning Phase).

1. **Estimated Marginal Means (EMMs)**

| **Sleep Treatment** | **Day** | **Emmean** | **SE** | **df** | **Lower CL** | **Upper CL** |
| --- | --- | --- | --- | --- | --- | --- |
| Interrupted | 1 | 2277.7 | 391 | 30 | 1478 | 3077 |
| Noninterrupted | 1 | 120.5 | 391 | 30 | -679 | 920 |
| Interrupted | 2 | 74.5 | 391 | 30 | -725 | 874 |
| Noninterrupted | 2 | 17.7 | 391 | 30 | -782 | 817 |
| Interrupted | 3 | 29.7 | 391 | 30 | -770 | 829 |
| Noninterrupted | 3 | 16.8 | 391 | 30 | -783 | 816 |

1. **Pairwise Comparisons (Sleep Treatment within Days)**

| **Day** | **Contrast** | **Estimate** | **SE** | **df** | **t-ratio** | **p-value** |
| --- | --- | --- | --- | --- | --- | --- |
| 1 | Interrupted - Noninterrupted | 2157.2 | 554 | 30 | 3.897 | 0.0005 |
| 2 | Interrupted - Noninterrupted | 56.8 | 554 | 30 | 0.103 | 0.9189 |
| 3 | Interrupted - Noninterrupted | 12.8 | 554 | 30 | 0.023 | 0.9817 |

**C. Pairwise Comparisons (Days within Sleep Treatment)**

| **Treatment** | **Contrast** | **Estimate** | **SE** | **df** | **t-ratio** | **p-value** |
| --- | --- | --- | --- | --- | --- | --- |
| Interrupted | Day1 - Day2 | 2203.167 | 546 | 20 | 4.034 | 0.0018 |
| Interrupted | Day1 - Day3 | 2248.000 | 546 | 20 | 4.116 | 0.0015 |
| Interrupted | Day2 - Day3 | 44.833 | 546 | 20 | 0.082 | 0.9963 |
| Noninterrupted | Day1 - Day2 | 102.833 | 546 | 20 | 0.188 | 0.9807 |
| Noninterrupted | Day1 - Day3 | 103.667 | 546 | 20 | 0.190 | 0.9803 |
| Noninterrupted | Day2 - Day3 | 0.833 | 546 | 20 | 0.002 | 1.0000 |

**Supplementary Statistics 4.** Multiple comparisons using Tukey method for Poisson generalised linear mixed model (GLMM) fitted for number of incorrect choices before food reward (Memory Phase).

1. **Estimated Marginal Means (EMMs)**

| **Sleep Treatment** | **Day** | **Emmean** | **SE** | **df** | **Asymp. LCL** | **Asymp. UCL** |
| --- | --- | --- | --- | --- | --- | --- |
| Interrupted | 1 | 0.826 | 0.281 | Inf | 0.2746 | 1.378 |
| Noninterrupted | 1 | 0.326 | 0.388 | Inf | -0.4339 | 1.086 |
| Interrupted | 2 | 1.021 | 0.258 | Inf | 0.5145 | 1.527 |
| Noninterrupted | 2 | 0.590 | 0.313 | Inf | -0.0229 | 1.203 |
| Interrupted | 3 | 0.672 | 0.302 | Inf | 0.0807 | 1.264 |
| Noninterrupted | 3 | 0.271 | 0.363 | Inf | -0.4401 | 0.983 |

1. **Pairwise Comparisons (Sleep Treatment within Days)**

| **Day** | **Contrast** | **Estimate** | **SE** | **df** | **z-ratio** | **p-value** |
| --- | --- | --- | --- | --- | --- | --- |
| 1 | Interrupted - Noninterrupted | 0.500 | 0.478 | Inf | 1.048 | 0.2946 |
| 2 | Interrupted - Noninterrupted | 0.431 | 0.403 | Inf | 1.069 | 0.2849 |
| 3 | Interrupted - Noninterrupted | 0.401 | 0.470 | Inf | 0.853 | 0.3934 |

**C. Pairwise Comparisons (Days within Sleep Treatment)**

| **Treatment** | **Contrast** | **Estimate** | **SE** | **df** | **z-ratio** | **p-value** |
| --- | --- | --- | --- | --- | --- | --- |
| Interrupted | Day1 - Day2 | -0.1942 | 0.360 | Inf | -0.539 | 0.8521 |
| Interrupted | Day1 - Day3 | 0.1541 | 0.393 | Inf | 0.392 | 0.9187 |
| Interrupted | Day2 - Day3 | 0.3483 | 0.376 | Inf | 0.925 | 0.6243 |
| Noninterrupted | Day1 - Day2 | -0.2640 | 0.484 | Inf | -0.545 | 0.8489 |
| Noninterrupted | Day1 - Day3 | 0.0545 | 0.518 | Inf | 0.105 | 0.9939 |
| Noninterrupted | Day2 - Day3 | 0.3185 | 0.464 | Inf | 0.686 | 0.7716 |

**Supplementary Statistics 5.** Multiple comparisons using Tukey method for linear mixed model (LMM) fitted for time to correct choice (Learning Phase).

1. **Estimated Marginal Means (EMMs)**

| **Sleep Treatment** | **Day** | **Emmean** | **SE** | **df** | **Lower CL** | **Upper CL** |
| --- | --- | --- | --- | --- | --- | --- |
| Interrupted | 1 | 13.83 | 3.63 | 22.7 | 6.314 | 21.4 |
| Noninterrupted | 1 | 7.28 | 3.93 | 25.0 | -0.809 | 15.4 |
| Interrupted | 2 | 21.17 | 3.63 | 22.7 | 13.648 | 28.7 |
| Noninterrupted | 2 | 8.67 | 3.63 | 22.7 | 1.148 | 16.2 |
| Interrupted | 3 | 8.83 | 3.63 | 22.7 | 1.314 | 16.4 |
| Noninterrupted | 3 | 7.83 | 3.63 | 22.7 | 0.314 | 15.4 |

1. **Pairwise Comparisons (Sleep Treatment within Days)**

| **Day** | **Contrast** | **Estimate** | **SE** | **df** | **t-ratio** | **p-value** |
| --- | --- | --- | --- | --- | --- | --- |
| 1 | Interrupted - Noninterrupted | 6.55 | 5.35 | 23.9 | 1.224 | 0.2328 |
| 2 | Interrupted - Noninterrupted | 12.50 | 5.14 | 22.7 | 2.434 | 0.0233* |
| 3 | Interrupted - Noninterrupted | 1.00 | 5.14 | 22.7 | 0.195 | 0.8474 |

**C. Pairwise Comparisons (Days within Sleep Treatment)**

| **Treatment** |  | **Contrast** | **Estimate** | **SE** | **df** | **t-ratio** | **p-value** |
| --- | --- | --- | --- | --- | --- | --- | --- |
| Interrupted |  | Day1 - Day2 | -7.333 | 4.04 | 19.0 | -1.816 | 0.1912 |
| Interrupted |  | Day1 - Day3 | 5.000 | 4.04 | 19.0 | 1.238 | 0.4461 |
| Interrupted |  | Day2 - Day3 | 12.333 | 4.04 | 19.0 | 3.054 | 0.0171* |
| Noninterrupted |  | Day1 - Day2 | -1.384 | 4.31 | 19.7 | -0.321 | 0.9449 |
| Noninterrupted |  | Day1 - Day3 | -0.550 | 4.31 | 19.7 | -0.128 | 0.9910 |
| Noninterrupted |  | Day2 - Day3 | 0.833 | 4.04 | 19.0 | 0.206 | 0.9768 |
